# HDAC6 and Ovarian Cancer

**DOI:** 10.3390/ijms14059514

**Published:** 2013-05-02

**Authors:** Joshua Haakenson, Xiaohong Zhang

**Affiliations:** 1 Department of Pathology and Cell Biology, University of South Florida Morsani College of Medicine, 12901 Bruce B. Downs Blvd., Tampa, FL 33612, USA; E-Mail: jhaakens@health.usf.edu; 2 Program of Molecular Oncology, H. Lee Moffitt Cancer Center and Research Institute, 12902 Magnolia Dr., Tampa, FL 33612, USA

**Keywords:** HDAC6, ovarian cancer, cancer-related signaling pathways, HDAC6 inhibitors, cell stress response, motility, oncogenesis, histone deacetylase

## Abstract

The special class IIb histone deacetylase, HDAC6, plays a prominent role in many cellular processes related to cancer, including oncogenesis, the cell stress response, motility, and myriad signaling pathways. Many of the lessons learned from other cancers can be applied to ovarian cancer as well. HDAC6 interacts with diverse proteins such as HSP90, cortactin, tubulin, dynein, p300, Bax, and GRK2 in both the nucleus and cytoplasm to carry out these cancerous functions. Not all pro-cancer interactions of HDAC6 involve deacetylation. The idea of using HDAC6 as a target for cancer treatment continues to expand in recent years, and more potent and specific HDAC6 inhibitors are required to effectively down-regulate the tumor-prone cell signaling pathways responsible for ovarian cancer.

## 1. Introduction

Histone acetyltransferases (HATs) and deacetylases (HDACs) have opposing effects on the acetylation status of their substrates, which include core histones and non-histone proteins. Thus, HDACs may be a misnomer since they deacetylate more substrates than just the histones in the nucleus. Either way, DNA wraps around histones in the nucleus. When HATs act on histones, gene expression levels tend to be up-regulated due to the increase in docking sites where transcription factors can bind. HDACs have a reverse effect [1]. Thus, HDACs take off an acetyl group from their substrate, including the histones of chromatin. Preserving the acetylation status in proteins by HDAC inhibitors has been shown to induce growth arrest and apoptosis of cancer cells *in vivo* and *in vitro*. Clinical trials show HDAC inhibitors to be effective anti-tumor drugs [2]. There are four classes of HDACs [3]. Class 1 includes HDACs related to the yeast gene *Rpd3*: specifically *HDAC1*, *HDAC2*, *HDAC3*, and *HDAC8*. Class 2 is related to the yeast gene *Hda1p* and includes *HDAC4*, *HDAC5*, *HDAC6*, *HDAC7*, *HDAC9*, and *HDAC10*. Class 3 includes the Sirtuin family of enzymes, *SIRT1*, *SIRT2*, *SIRT3*, *SIRT4*, *SIRT5*, *SIRT6*, and *SIRT7*. Class 4 is only made up of *HDAC11*. Class 2 HDACs are further divided into Class 2a, which includes *HDAC4*, *HDAC5*, *HDAC7* and *HDAC9*, and class 2b, which includes *HDAC6* and *HDAC10*. One unique characteristic of the HDACs is their ability to shuttle between the cytoplasm and the nucleus. Because of this shuttling capacity, it was soon discovered that they act on more than just histones [4]. HDACs can be part of transcription corepressor complexes. More specifically, class 2 HDACs have been implicated in cell differentiation and development [5].

HDACs catalyze the deacetylation of lysine residues in the *N*-terminal tails of histones and other substrates [6]. Nucleosomes are the basic unit of the interaction between histone proteins and nucleic acids. Acetylation of histones changes the conformation of this interaction, thus altering the level of gene expression [7]. Lysine acetylation has been found to be very important in prokaryotic energy metabolism since a group from Texas found 138 acetylation sites in 91 proteins. 70% of these proteins were found to be metabolic enzymes and translation regulators [8]. A group from Germany used mass spectrometry to find 3600 lysine acetylation sites on 1750 proteins, mainly involved in large macromolecular complexes involved in the cell cycle, splicing, nuclear transport, actin nucleation and chromatin remodeling [9].

HDAC6 is unique in having two deacetylase domains. One report showed that both of the deacetylase domains are required for its deacetylase activity [10]. This report used HDAC6 immune complexes purified from 293T cells [10]. Changing the linker region between the two domains decreased their action [10]. Another report used highly purified recombinant HDAC6 and site-directed mutagenesis to assess the relative necessity in the deacetylase reaction of the two histidine sites residing in the *N*-terminal and *C*-terminal deacetylase domain [11]. A histidine (H) to alanine (A) mutation at amino acid residue 216 slightly lowered the catalytic rate, but H611A lowered the catalytic rate greater than 5000 times [11]. Thus, the *C*-terminal deacetylase domain is highly active *in vitro* [11]. HDAC6 also has an ubiquitin-binding zinc finger [12,13], a nuclear localization signal, a nuclear export signal, and a tetradecapeptide repeat domain [14]. The unbiquin-binding zinc finger of HDAC6 binds to mono- and polyubiquitin as well as the ubiquitinated proteins [12,13,15]. The zinc finger domain is critical for HDAC6’s functions. For example, HDAC6 can bind to polyubiquitnated misfolded proteins by its zinc finger and transport them to the aggresome [15]. Thus, HDAC6 is an important player in the misfolded protein-induced stress. More recently, HDAC6 was also found to govern the stability of the cellular pool of ubiquitinated protein via its ubiquitin-binding activity [16]. The tetradecapetide repeat domain of HDAC6 presents only in the human ortholog, but not in the mouse. Due to these structural difference, human but not murine HDAC6 would not translocate to the nucleus upon leptomycin B (LMB) treatment [14,17]. Therefore, the tetradecpetide repeat domain plays an essential role in retaining human HDAC6 in the cytoplasm.

HDAC6 is a cancer drug target because of its role in transforming normal cells to cancer cells. Excess HDAC6 is associated with tumorigenesis and cell survival; therefore, HDAC6 can be used as a marker for prognosis. In multiple myeloma cells, blocking expression of HDAC6 can cause apoptosis. In breast cancer MCF-7 cells, HDAC6 helps lead to metastasis by up-regulating cell motility [17]. HDAC6 also interacts with cortactin, which also helps regulate cell motility [18].

## 2. Ovarian Cancer

The average age of diagnosis for ovarian cancer is 63, and there were 22,280 new cases in 2012. As of 1 January, 2012, there were 192,750 women alive who had been diagnosed with ovarian cancer. The American Cancer Society estimates 14,030 deaths from ovarian cancer in 2013 [19].

Treating ovarian cancer can cause many side-effects including a decrease in bone density, cardiovascular diseases, cognitive defects, fear of cancer recurrence, distress, pain and infertility [20]. Common treatment of ovarian cancer is surgery and chemotherapy. Some of the drugs used on the disease are doxorubicin hydrochloride, carboplatin, cisplatin, cyclophosphamide, gemcitabine hydrochloride, topotecan hydrochloride, and paclitaxel [21].

Ovarian neoplasms can arise from the surface epithelium, the gonadal or sex-cord stroma, germ cells, fallopian tubes or from metastasis from other tumors [22]. Endometrioid ovarian cancer can arise from activation of K-ras and deletion of PTEN [23]. Ovarian cancers can also arise from mutations in *p53*, *BRAF*, *β-catenin*, *Rb*, *BRCA1*, *BRCA2*, *MSH2* and *MLH1* and overexpression of *epithelial growth factor receptor* (*EGFR*), *Akt* and *HER2*. HDAC inhibitors are promising drugs for ovarian cancer treatment, and there are several currently in clinical trials. Epithelial ovarian cancers, including serous, mucinous, clear cell, and endometrioid, constitute 80%–90% of ovarian cancers [24]. In addition to the molecular mechanisms hinted at above, these neoplasms are also under endocrine regulatory mechanisms by gonadotropin-releasing hormones, gonadotropins, estrogen, progesterone, and androgens, and thus there are hormonal therapies for ovarian cancer as well [25]. Recently, we found that HDAC6 protein levels are elevated in a panel of ovarian cancer tissue samples compared with the benign samples (our unpublished data), suggesting that HDAC6 may play a critical role in ovarian cancer development.

## 3. HDAC6 Levels in Cancer Cell Lines

In many murine cell lines (Balb/c3T3, B16, MEL, and FM3A) there is much more HDAC6 in the cytoplasm than in the nucleus. During cell differentiation and proliferation in B16 cells, many HDAC6 proteins are found to translocate to the nucleus as well [26]. MCF-7 breast cancer cells stimulated with estrogen show up-regulation of *HDAC6* gene expression [27]. *HDAC6* is located on the Xp11 chromosome, and HDAC6 seems to be found in the brain, breast, colon, ovary, pancreas, prostate and heart, and may be up-regulated in cancers of the brain, breast, ovary, and pancreas [28]. Additionally, there may be a longer disease-free survival in patients with high expression levels of *HDAC6* because they can be more susceptible to HDAC inhibitor treatment [29]. Greater levels of *HDAC6* were found in oral squamous cell carcinomas (OSCC) than in the normal oral keratinocytes (NOKs). The cell lines investigated included Ca9-22, OK92, Ho1-N-1, HSC2, HSC3, HSC4, SAS, and Sa3 [30]. Acute myeloid leukemia samples and leukemic cell lines, including HL60, K562, and KG1a also showed increased levels of HDAC6 [31].

## 4. Oncogenesis

Measuring mouse embryonic fibroblasts (MEFs) in an anchorage-independent fashion, researchers from Duke found that HDAC6 is required for oncogenesis [32]. They also looked at malignant transformations in cancer cells such as SKOV3, SKBR3, and MCF7, and anoikis in the SKOV3 cell [32]. Later groups looked at the mechanisms by which this occurs. Survivin in the cytoplasm decreases apoptosis through inactivating caspase proteins. Acetylation by CREB-binding protein (CBP) makes survivin translocate to the nucleus, where it binds to STAT3, thus inhibiting STAT3 from increasing gene expression activity in the nucleus [33]. HDAC6 deacetylates survivin and so can increase survivin levels in the cytoplasm, thereby activating oncogenesis [34]. Combining farnesyltransferase inhibitor lonafarnib and paclitaxel inhibits the oncogenic activity of HDAC6 [35].

## 5. Cellular Stress Response

A link was discovered between protein acetylation and ubiquitination when HDAC6 was found to interact with proteins of the ubiquitin signaling pathway, p97/VCP/Cdc48p and phospholipase A2-activating protein. HDAC6 has a zinc-finger ubiquitin-binding domain, which can bind a mono- or polyubiquitin and ubiquitinated proteins [36,37]. Abnormalities in the ubiquitin system can lead to ovarian cancer pathology caused by the cell-cycle, signal transduction cascades, transcriptional regulators, and endocytosis malfunction [12]. HDAC6 has also been copurified with deubiquitinating enzymes and can bind polyubiquitin [36,37]. HDAC6 also interacts with HSP90, showing that it has an important role in stress response [13]. When researchers inhibited VCP using RNAi in HeLa cells, dispersed aggregates did form, but they were noted as distinct from aggresomes [38]. An investigation of the zinc-finger ubiquitin-binding domain of HDAC6 showed its functional consequences to down-regulate polyubiquitin chain turnover, leading to an increase in polyubiquitinated proteins. P97/VCP/Cdc48p counteracts this response [39]. HDAC6 is also intimately involved with G3BP (Ras-GTPase-activating protein SH3 domain-binding protein 1) and so affects the formation of stress granules of the stress response [16]. Stress granules increase when the ubiquitin-proteasome pathway is depleted [40]. Tax is a human T cell leukemia virus type-1 (HTLV-1) protein. Tax binds to HDAC6 and so can inhibit stress granule formation [41].

When the proteasome is inhibited, the E3-ubiquitin ligase TRIM50 can send polyubiquitinated proteins to an aggresome for storage. This action is mediated by HDAC6 and p62 [42]. An aggresome is a toxic aggregate of proteins that can cause many diseases. Aggresome formation is prevented by molecular chaperones or proteasomal degradation [43]. Molecular chaperones can help refold misfolded proteins; the proteasome degrades other misfolded proteins. When an aggresome forms, it can be shuttled along microtubule tracks to the microtubule organizing center (MTOC) by dynein. Molecular chaperone and proteasome proteins are constantly attaching and detaching from the aggresome to resolve the problem of misfolded proteins. Many chaperone proteins have been identified, including HSC70, heat shock protein 40 (HSP40), HSP70, and HSP90 [44]. The aggresome can also be targeted to lysosomes in the process of autophagy [45]. Proteins associated with the aggresome include p97/VCP/Cdc48p, 14-3-3 and Bmh1 [46]. HDAC6 is also associated with aggresomes. It can bind to the polyubiquitinated misfolded proteins and the dynein motors, acting as a link between them as they are moved to the aggresomes [15,47]. Another function of HDAC6 in this stress response is to activate heat shock factor 1 (HSF1) by making it dissociate with its repressor, HSP90. The dissociated HSF90 acts as a molecular chaperone helping in the refolding of misfolded proteins. In these two ways HDAC6 can aid in resolving the cytotoxicity of aggresomes [48]. The protein kinase CK2 phosphorylates HDAC6, increasing HDAC6’s activity in the cellular stress response [47].

After the aggresomes are formed, HDAC6 also plays a role in their clearance by autophagy, which leads to degradation by lysosomes. When the cell was stressed with MPP(+) (1-methyl-4-phenylpyridinium) and HDAC6 was silenced, aggresome formation and autophagy were decreased [49]. Sequestosome 1/p62 also promotes polyubiquitinated protein degradation by shuttling them to the proteasome [50]. Because the autophagic marker light chain 3 (LC3) was found bound to sequestosome 1/p62, it was suggested that sequestosome 1/p62 also plays a role in connecting aggresomes to autophagy [51]. Modulating autophagy is important in tumor cells [52]. Autophagy is essential to developing cells as well, but too much would result in cell death, so a fine balance must be reached in healthy tissues [53]. HDAC6 is responsible for directing misfolded proteins to autophagy when the ubiquitin proteasomal system is not working, especially in the neurodegenerative diseases [54]. Autophagy became more complex when it was found that different molecular mechanisms underlie nutrient-regulated autophagy (which aids in maintaining homeostasis and breaking down nutrients into macromolecules the cell can use again) versus quality-control autophagy (which breaks down toxic aggresomes accumulating from the misfolded protein aggregates). HDAC6 plays a role in both [55]. In quality-control autophagy, it certainly regulates the fusion of autophagosomes to lysosomes, in conjunction with the actin cytoskeleton, including cortactin [56]. If the above-mentioned ubiquitin proteasome degradation pathway is down-regulated, autophagy may increase [57]. When the proteasome was inhibited by bortezomib in MCF-7 breast cancer cells, autophagy was induced; when HDAC6 was knocked down, autophagy decreased as well [58]. Other work shows that HDAC inhibition induces autophagy in cancer cells [59].

As aforementioned, molecular chaperones can counterbalance the proteasome as the cell finds the middle ground for its protein levels [60]. One major chaperone is HSP90, regulated by acetylation and HDAC6 deacetylation [61]. Knockdown of HDAC6 leads to hyperacetylation of HSP90 in human embryonic kidney cells, and hence HSP90’s inability to properly fold the glucocorticoid receptor (GR) [62]. Disabled GR can affect social function and stress-response [63]. In C4-2 castration-resistance prostate cancer cells, HDAC6 seemed to regulate the androgen receptor (AR), also through deacetylation of HSP90 [64]. Inhibiting HDAC6 with hydroxamic acid (HAA) analogues LAQ824 and LBH589 increased acetylation of HSP90 and decreased its activity, allowing its client proteins, such as Bcr-Abl, c-Raf and AKT, to be polyubiquitinated in human leukemia cells [65]. Two more interesting stress proteins are heat-shock protein 27 (HSP27) and HSF1, mentioned earlier with aggresomes. Mutations in HSP27 can cause Charcot-Marie-Tooth disease [66]. When *RAS* or *p53* is mutated, HSF1 can help protect mice from developing tumors. In cells, however, HSF1 may play a supportive role in tumorigenesis through proliferation, survival, and protein synthesis mechanisms. Indeed, some cancer cell lines seem to be at least partially dependent on HSF1 function [67].

## 6. Motility

HDAC6 also plays an important role in cell migration and mobility through its action on the cytoskeleton [68]. It has long been known that microtubules are important in the cytoskeleton remodeling that takes place during migration [69], and the cell polarity required for sustained motility [70]. Microtubules are also necessary for cell division [71]. In 1987, they were found to contain acetylated α-tubulin [72]. A decade and a half later, HDAC6 was found to be a microtubules deacetylase that could act on these tubulin subunits, thus regulating cell motility [73]. Interestingly another member of the HDAC family also acts as a tubulin deacetylase, SIRT2 [74]. It is the balance of acetylation and deacetylation that controls microtubule dynamics [75]. Overexpression of HDAC6 leads to tubulin hypoacetylation and thus greater cell motility [76]. Inhibiting HDAC6 with TSA created increased microtubule acetylation and decreased microtubule dynamics, and thus decreased cell mobility [77]. Cell adhesion turnover, also important in cell motility, decreased significantly with HDAC6 inhibition [78]. Furthermore, HDAC6 has been found abundant in the testes, but testes develop normally without HDAC6. It seems to play a bigger role in bone development, where decreased HDAC6 results in increased cancellous bone mineral density [79]. Another in-depth investigation found the Rho-mDia2-HDAC6 pathway responsible for partially controlling osteoclast maturation [80]. Combining the facts that HDAC6 binds dynein motors and controls microtubule formation, researchers from Duke found that HDAC6 has a role in intracellular organelle trafficking, specifically by interacting with the EGFR [81]. This trafficking may rely on kinesin-1 [82]. Failure to function of microtubule-based transport can result in neuronal toxicity and Huntington’s disease [83]. Finally, polymerizing microtubules are important in blood platelet shape [84].

The cytoskeleton is partially made up of microtubules and actin filaments. In addition to microtubules, as it turns out, HDAC6 also deacetylates cortactin, a protein that binds to actin [18,85], F-actin, specifically. Cortactin is about 80–85 kilodaltons. It is localized in the cortex of the cell, hence the name cortactin [86]. Cortactin assembly and disassembly helps the cell migrate, with the lamellipodia protruding towards the direction of movement. Cells without cortactin have handicapped invasion and motility [87]. Most importantly, HDAC6 deacetylates cortactin. This mediates a change in the cell motility dependent on actin filaments, similar to HDAC6’s influence on microtubule-associated movement [18]. In addition to motility, one functional consequence of HDAC6 deacetylating cortactin appears to be an effect on angiogenesis in endothelial cells [88]. Along with HDAC6, SIRT2 also induces migration, invasion and metastasis in bladder cancer [89].

Other proteins involved with cell motility include dysferlin, GRK2, and calpain. Dysferlin has an inhibitory interaction with HDAC6, leading to increased tubulin acetylation [90]. With calcium present, dysferlin also binds phosphatidylserine, phosphatidylinositol 4-phosphate, and phosphatidylinositol 4,5-bisphosphate, which may or may not be important in cellular motility [91]. G protein-coupled receptor kinase 2 (GRK2) phosphorylates HDAC6, increasing HDAC6 deacetylase activity on tubulin, which leads to increased cell motility [92]. GRK2 is also regulated by phosphorylation [93]. Calpain requires calcium to increase cell motility; it is another potential target for metastasis inhibitors [94].

Cell motility is used as a model for studying cancer metastasis [95]. It seems there is crosstalk between the microtubule and actin proteins involved in this motility [96]. HDAC6 may be significant. HDAC6 is significant in MCF-7 breast cancer cells. When these cells overexpressed HDAC6, cell motility was found to drastically increase [97]. Complicating motility mechanisms, HDAC6 interacts with breast cancer metastasis suppressor 1 (BRMS1). BRMS1 is stabilized by HSP90, and HSP90 is a target of HDAC6. This triangle of interactions may provide an important target for breast cancer metastasis inhibition [98]. Another study has found that HDAC6 is important in MDA-MB-231 cells. HDAC6 inhibition in these cells by siRNA or TSA down-regulated their invasive ability in a 3D type 1 collagen matrix [99]. Similar results were found in hepatocellular carcinoma [100]. Lastly, HDAC6 has been shown to control blood platelet spreading, a process necessary for hemostasis following injury to blood vessels [101].

## 7. Cancer-Related Signaling

So far we have explored several signaling pathways that lead to cancer. There are many more, some including Ku70, Tat, and CYLD. Ku70 may be the most important in neuroblastomas [102]. It is involved in DNA repair mechanisms and also can suppress the tumor suppressing action of apoptosis through its interaction with Bax [103]. CREB-binding protein (CBP) acetylates Ku70, and HDAC6 deacetylates it, thus controlling its binding with Bax. When Ku70 is acetylated, it releases Bax and apoptosis can occur. Deacetylated Ku70 remains bound to Bax, preventing apoptosis and leading to cancer [104]. The human immunodeficient virus (HIV) trans-activator protein, Tat, present in transcriptional augmentation, is also regulated by the acetylation/deacetylation mechanism. p300 and p300/CBP-associating factor (PCAF) acetylate Tat [105]. HDAC6 deacetylates Tat [106]. CYLD is a tumor suppressor mutated in a skin cancer called familial cylindromatosis [107]. Part of its function comes from inhibiting NFκB. Snail can inhibit CYLD expression in melanoma [108]. CYLD controls cell-cycle progression by interacting with Bcl-3 and delaying the transition from G1 to S phase. CYLD also inhibits HDAC6, leading to increased acetylated tubulin and less cellular motility, as we saw before [109]. Surprisingly, NFκB binds to HDAC6 as well, suppressing H(+)-K(+)-ATPase α(2) expression. p50 and p65 also bind to HDAC6 [110].

Many more HDAC6-related proteins remain. One such protein is Runx2, the first transcription factor found to bind to HDAC6. HDAC6 regulates the activity of Runx2 by repressing its activation of p21 [111]. Also, peroxiredoxins (Prxs), in addition to their antioxidant function, can control hydrogen peroxide influence on signal transduction cascades [112]. HDAC6 deacetylates peroxiredoxins [113]. Ligand-dependent corepressor (LCoR) is a repressor of transcription [114]. HDAC6 colocalizes with LCoR, and the two together may have both corespressor and activator activities depending on their target genes [115]. Cilia assembly and disassembly is partially policed by phosphorylated HDAC6, itself activated by an interaction between the Aurora A kinase (AurA) and the prometastatic scaffolding protein HEF1/Cas-L/NEDD9. Inhibiting HDAC6 and Aurora A stabilize cilia [116]. The aberrant Wnt signaling pathway can cause cancer. Either Wnt ligands by themselves, or epidermal growth factor (EGF) inducing HDAC6 to deacetylate beta-catenin, can cause beta-catenin nuclear localization, which can upregulate oncogenes such as c-Myc [117]. It seems that HDAC6, protein kinase C alpha (PKCα), and beta-catenin are all involved in the induction of type I interferon (IFN) transcription that occurs when the cell becomes infected with a cancer virus. IFN can also be induced by the transcription factors interferon regulatory factor (IRF3) and nuclear factor κB (NF-κB) [118]. It is worthwhile to keep in mind that p300 can acetylate HDAC6, causing a decrease in HDAC6’s deacetylating activity [119]. This action of p300 can also modulate HDAC6 nuclear import by blocking the HDAC6/importin-α interaction [120]. HDAC6 is also a part of the Akt-GSK3beta signaling pathway that modulates mitochondrial transport. GSK3beta may phosphorylate HDAC6 [121]. HDAC6 also influences neuropathies caused by mutations in HSPB-1 through its acetylation of tubulin [122].

## 8. HDAC6 Inhibitors

Several lines of evidence suggest that HDAC6 is an ideal target for cancer therapy. First, mice lacking HDAC6 display hyperacetylated tubulin in many tissues tested but are viable and develop normally [79]. Second, *HDAC6* knockout MEFs are resistant to transformation [32]; *HDAC6* knockout mice might be less prone to cancer. Third, normal microtubule dynamics are essential for cellular functions; microtubule acetylation will disturb such functions. Several chemotherapeutic agents such as paclitaxel, which targets microtubules, are currently used to treat cancer patients. Based on the above information, one would expect that inhibition of HDAC6 would exert minimal side-effects and can effectively augment the current anti-tumor drugs. Indeed, researchers have been working very hard at inhibiting HDAC6 activity selectively and effectively. Early pan-HDAC inhibitors include trichostatin A (TSA) and suberolyanilide hydroxamic acid (SAHA). These inhibitors can inhibit cell growth and prevent the formation of tumors in mice models [123]. Another inhibitor was found after a multidimensional, chemical genetic screen of 7392 small molecules. This molecule was called tubacin, and was found to inhibit HDAC6 deacetylase activity, especially on tubulin, without altering histone acetylation and cell-cycle progression [124]. This same chemical genetic screen also found histicin, a less-successful histone inhibitor [125]. Hydroxamate inhibitors seem to work for many of the HDACs [126]. Acting either directly or indirectly, the combination of lonafarnib and paclitaxel also inhibits the deacetylating activity of HDAC6 and its metastatic tendencies [35]. When tubacin was combined with the proteasome inhibitor bortezomib, there was even more significant anti-tumor inhibition [127]. HDAC6 small interfering RNA was also used with bortezomib to show increased inhibition of apoptosis in pancreatic cancer cells [128]. Tubulin binds and inhibits the SIRT2/HDAC6 complex [129].

The search for potent HDAC6 inhibitors to block cancer cell proliferation continued strong through 2007. Many pan-HDAC inhibitors had been found by this time, and researchers were becoming more interested in less-toxic, more-selective, single isoform inhibitors to more deftly regulate the development of oncocells [130]. A few thiolate analogues with bulky alkyl and tert-butylcarbamate groups showed effective HDAC6 and cancer cell growth inhibition [131]. Mercaptoacetamides showed potential for HDAC6 inhibition [132]. A triazolylphenyl-based compound with an active phenyl group showed great promise against HDAC6 [133]. Nitrile oxide cycloaddition was used to find another hydroxamate inhibitor containing a phenylisoxazole group [134]. The combination of bortezomib and SAHA caused cell death in multiple myeloma cells through a Myc-Noxa mediation [135]. This combination therapy of inhibiting both the proteasome and HDAC6 also seemed to work well in ovarian cancer cells with the treatment of bortezomib and the HDAC6-specific inhibitor NK84. The mechanism of this inhibition is likely that bortezomib causes increased ubiquitin-proteasome-system stress, and HDAC6 is unable to clear the aggresomes to the lysosomal pathway, thus causing cell death [136]. Hydroxamic acids with a pyridylalanine substructure were also found to be effective in inhibiting HDAC6 [137]. In 2009, the HDAC6 inhibitors 3,4-dihydroquinaxalin-2(1*H*)-one and piperazine-2,5-dione aryl hydroxamates were synthesized [138].

Still in 2009, clinical trials found many side-effects associated with these inhibitors, and so the search continued for the magic bullet [139]. A good therapeutic index was found with biphenyl-4-yl-acrylohydroxamic acid in Italy [140]. A group in California used cyclic tetrapeptides in their quest [141]. A Canadian group used non-natural macrocyclic inhibitors [142]. A naphthoquinone structure was found to inhibit HDAC6 in 2012 [143]. One limiting factor in this search is that there is no three-dimensional crystal structure for HDAC6 in the human [144]. Researchers still continue their work in the effort to manipulate cell death and the cell cycle, and hence cancer, through inhibition of HDAC6 [145].

## 9. Conclusions

Recently, HDAC inhibitors have shown promise as agents against ovarian cancer. In 2007, a group found that the class 1 HDAC inhibitor, R306465, worked well in preventing the growth of A2780 ovarian, H460 lung, and HCT116 colon carcinomas [146]. In 2008, it was found that HDAC6 is important in tumorigenesis [32]. In the same year, bortezomib and an HDAC6 inhibitor, NK84, were found to kill ovarian cancer cells [136]. In 2010, SAHA and paclitaxel were found to be a potent combination against ovarian cancer [147]. Apoptosis and ovarian cancer cell death were upregulated with a C6-ceramide, TSA combination [148]. Thailandepsins also seem to be powerful against this cancer [149]. Just last year, tubastatin A, an HDAC6-selective inhibitor, showed strong anti-ovarian cancer tendencies [150], and the same thailandepsins along with romidepsin showed promising inhibition of ovarian cancer DNA damage response pathways [151].

**Figure 1:**
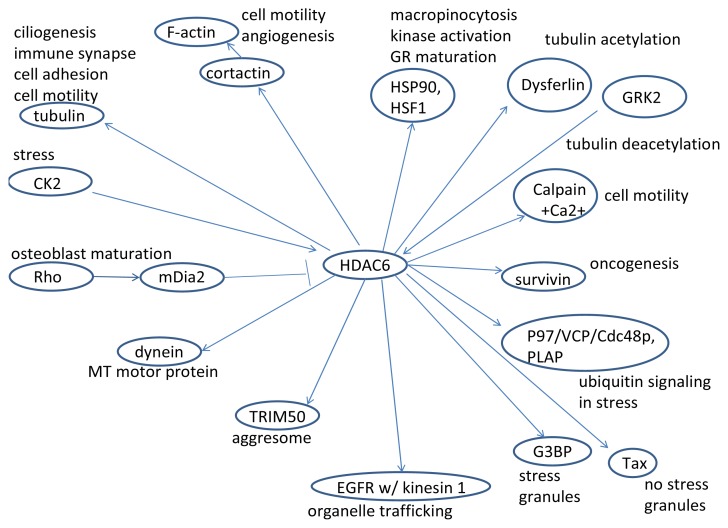
HDAC6 is involved with tubulin, cortactin, HSP90, HSF1, mDia2, Dysferlin, GRK2, Calpain, survivin, p97/VCP/Cdc48p, PLAP, dynein, CK2, TRIM50, EGFR, G3BP and Tax, and their functional consequences.

**Figure 2:**
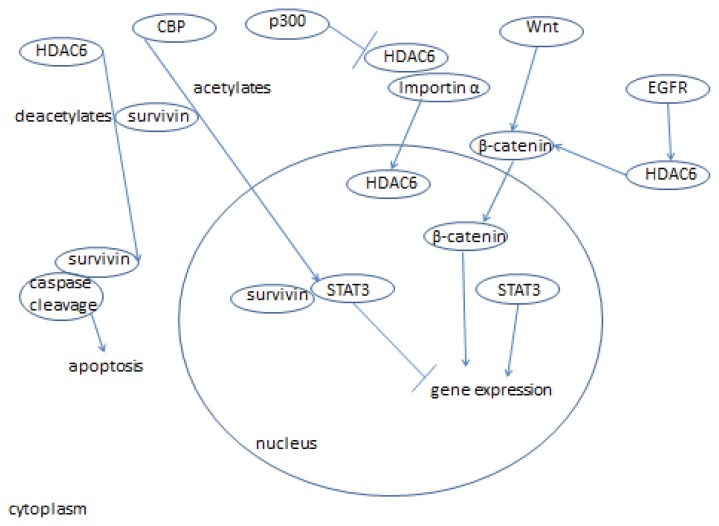
HDAC6 interacts with EGFR, survivin, and β-catenin, and their downstream functional consequences.

**Figure 3:**
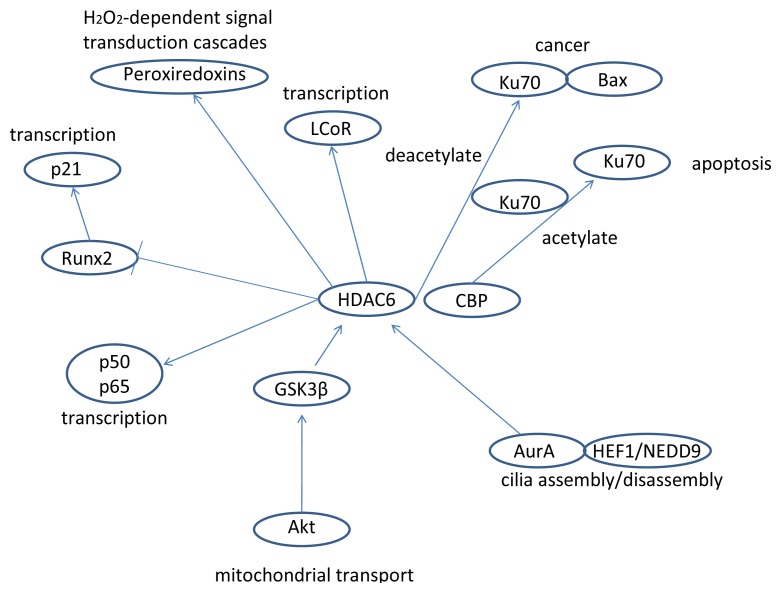
The interactions of HDAC6 with peroxiredoxins, LCoR, Ku70, GSK3β, Aurora A, p50, p65, and Runx2 and some functional consequences.

**Figure 4:**
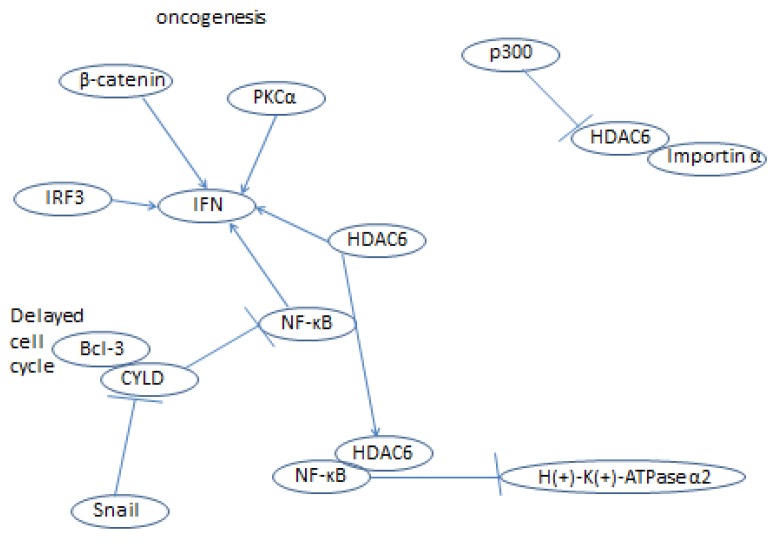
HDAC6 interacts with p300, importin α, IFN, and NF-κB, and their related processes.

**Figure 5:**
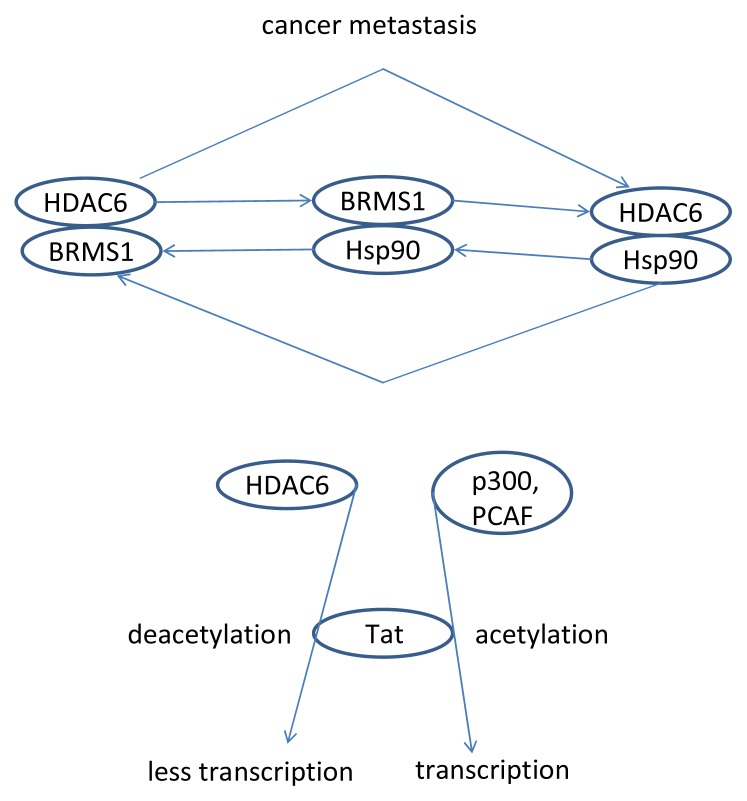
HDAC6’s interaction with Tat, and the triangle of interactions among HDAC6, BRMS1, and Hsp90.

As we have seen, HDAC6 plays a role in many cellular processes; it is one of the most important histone deacetylases in the cytoplasm and also controls interesting processes in the nucleus (HDAC6 signaling is summarized in Figures 1, 2, 3, 4 and 5) [152]. HDAC6 does influence the pathways of ovarian cancer through its effects on the stress response, oncogenesis, cell motility, and many other cancer-related signaling networks. Extensive use of HDAC inhibitors have elucidated mechanisms of cancer cell growth, development and metastasis, and this line of work continues to be pursued leading, hopefully, to a cure for cancer and alleviation of thousands of patients’ suffering.
